# Analysis of Natural Killer cell functions in patients with hereditary hemochromatosis

**DOI:** 10.17179/excli2020-1116

**Published:** 2020-03-25

**Authors:** Vivian Bönnemann, Maren Claus, Barbara Butzeck, Daniela Collette, Peter Bröde, Klaus Golka, Carsten Watzl

**Affiliations:** 1 Department for Immunology, Leibniz Research Centre for Working Environment and Human Factors (IfADo) at TU Dortmund, Dortmund, Germany; 2 Hämochromatose-Vereinigung Deutschland e.V. HVD, European Federation of Associations of Patients with Haemochromatosis EFAPH, Hattingen, Germany; 3 Gemeinschaftspraxis für Hämatologie und Onkologie, Dortmund, Germany

**Keywords:** hereditary hemochromatosis, immune system, iron, Natural Killer Cells

## Abstract

Hereditary hemochromatosis (HH) is an autosomal-recessive disorder of the iron metabolism. Patients are typically affected by dysregulated iron levels, which can lead to iron accumulation within essential organs, such as liver, heart and pancreas. Furthermore, many HH patients are also afflicted by several immune defects and increased occurrence of autoimmune diseases that are linked to human homeostatic iron regulator protein (HFE) in the immune response. Here we examined immune cell phenotype and function in 21 HH patients compared to 21 healthy controls with a focus on Natural Killer (NK) cells. We observed increased basal and stimulated production of pro-inflammatory cytokines such as IL-1β or IL-18 in HH patients compared to healthy controls. However, we did not find major changes in the phenotype, the amount or the cytotoxic function of NK cells in HH patients. Instead, our data show a general decrease in the total number of granulocytes in HH patients (2774 ± 958 per μl versus 3457 ± 1122 per μl in healthy controls). These data demonstrate that NK cells of HH patients are not significantly affected and that the patients' treatment by regular phlebotomy is sufficient to avoid systemic iron overload and its consequences to the immune system.

## Introduction

Although iron plays a crucial role for cell functionality, it is also known to generate toxic reactive oxygen species (ROS) that can harm the organism. Accordingly, iron concentrations are tightly regulated, both systemically and intracellularly (Porto and De Sousa, 2007[24]). This regulation is important for the iron metabolism of a broad range of organisms. At the same time, a host organism can modulate its iron levels in order to make iron less available for pathogenic microorganisms and for the generation of reactive oxygen species to combat unwanted invaders (Radtke and O'Riordan, 2006[26]; Weinberg, 2005[33]). Hence, the immune defense is inevitably linked to the iron metabolism.

Connections between iron overload and the immune system were already described for diverse immune cell types, such as abnor-mal ratios of T cell subsets in hemochromatosis patients (de Sousa et al., 1991[12]; Porto and De Sousa, 2007[24]). Hereditary hemochromatosis (HH) is an autosomal-recessive disorder in which the iron metabolism is dysregulated, leading to a systemic iron overload (Adams et al., 2018[1]; European Association for the Study of the Liver, 2010[13]). Accumulation of high levels of iron can lead to an irreversible destruction of essential organs such as the heart, liver and pancreas. In HH patients the systemic iron overload is caused by a mutation of the iron sensor protein HFE (Human homeostatic iron regulator protein). HFE and transferrin receptor 1 (TfR1) are triggering a signaling cascade to regulate hepcidin, which is a systemic regulatory hormone of the iron metabolism. Hepcidin acts as a ligand for ferroportin, an iron exporter expressed on the surface of macrophages, hepatocytes and the basolateral surface of enterocytes. Binding of hepcidin to ferroportin results in the internalization and degradation of the receptor, thereby blocking iron efflux from cells. Via this mechanism, HFE regulates iron absorption in the small intestine as well as iron recycling by macrophages (Powell and Yapp, 2000[25]; Reuben et al., 2017[28]). In the case of hereditary hemochromatosis, the mutated HFE is unable to fold properly or undergo posttranslational processing and is therefore targeted for degradation, resulting in hepcidin deficiency and heightened iron entry into the bloodstream (Barton et al., 2015[5]; Feder et al., 1997[15]; Reuben et al., 2017[28]). Therefore, HFE plays an important role in the iron metabolism. 

Due to its remarkable structure similarity to MHC I molecules, HFE was originally named human leukocyte antigen (HLA)-H, which underlines again the connection between the iron metabolism and the immune system (Feder et al., 1996[14]). HFE is categorized as a non-classical MHC Ib molecule and although it does not bind peptides, HFE is recognized by T cells. Imbalanced CD4/CD8 ratios of T cells were found in hemochromatosis patients with HFE mutations. Additionally, the non-classical MHC Ib molecules such as HLA-E, -F and -G have been shown to bind to receptors on Natural Killer (NK) cells with effects on immunoregulation, autoimmunity and immune tolerance during pregnancy (Hamerman et al., 2005[20]; Reimao et al., 1991[27]).

NK cells belong to the innate immune system and are very important for early immune reactions against viruses, malignant cells or parasites (Watzl, 2014[31]; Watzl et al., 2014[32]). NK cells are found in peripheral blood, representing 5-15 % of all lymphocytes. Additionally, they are found in tissues such as spleen, bone marrow, liver, uterus, or lungs (Bryceson et al., 2006[8]). The activity of NK cells is regulated by the interplay of activating and inhibitory signals, originating from a large array of different surface receptors. Human NK cells are characterized as CD56^+^, NKp46^+^, CD3^-^ cells that can kill infected or transformed cells by the release of granzyme and perforin filled granules or via the activation of death receptors on their target cells (Cooper et al., 2001[11]; Fehniger et al., 2003[16]; Pegram et al., 2011[23]). Activation of NK cells also results in the production of cytokines such as IFN-γ, which is important for the regulation of adaptive immune responses. 

Due to the fact that imbalanced CD4/CD8 T lymphocyte ratios were already described for HH patients we took a closer look at the influence of the iron metabolism on the immune system. In our preliminary studies some HH patients showed aberrant NK cell numbers and activities. Therefore, we decided to focus our analysis on NK cells.

## Materials and Methods

### Patients

Participants included in this study signed the informed consent. Participants described as patients in this work were diagnosed with hereditary hemochromatosis. Only age and gender of participants are known, while date of diagnosis, type of HFE mutation and other diseases or infections are unknown. This study includes a group of 21 patients with a mean age of 59.6 years and a male proportion of 52.4 %. The age-matched healthy control group (n=21) has a mean age of 56.3 years and a male proportion of 42.9 %.

### Cell culture

The K562 chronic myeloid leukemia cell line was maintained in IMDM medium (Life technologies^TM^) supplemented with 10 % fetal bovine serum (Invitrogen^TM^) and 10 Units/ml Penicillin / 10 μg/mL Streptomycin (Life technologies^TM^).

### PBMC isolation

Peripheral blood mononuclear cells (PBMC) were isolated by Ficoll density centrifugation from heparinized whole blood. PBMCs were stored at -170 °C until used for experiments.

### Absolute cell count

To determine the absolute number of leukocyte populations, 50 μl fresh heparinized blood sample was added to TruCount tubes (BD Biosciences) and incubated for 20 min at RT with the indicated antibodies (Table 1(Tab. 1)). Samples were subjected to erythrocyte lysis using FACS Lysing Solution (BD Biosciences, Heidelberg, Germany) and were measured on a BD LSRFortessa (BD Biosciences). Data were analyzed using FlowJo Software (FlowJo LLC, USA). Absolute cell number in blood samples was calculated as


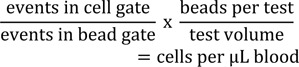


### Multicolor flow cytometry

For immunophenotyping three different panels were set up to analyze the samples for (1) a general leukocyte overview, (2) NK and T cell activation and memory markers and (3) activating NK cell receptors. All applied antibodies and dilutions are listed in Table 1(Tab. 1). Samples were thawed and kept on ice during the staining. For each panel 2 x 10^6^ PBMC were first stained with zombie yellow for 20 minutes in the dark. After a wash step with FACS buffer (DPBS, Gibco^® ^+ 2 % fetal bovine serum, Gibco^®^) samples were stained with the individual antibody mixture for 20 minutes in the dark. Cells were resuspended in 150 μl FACS buffer and kept on ice until analysis at the same day on a BD LSRFortessa. Data were analyzed using the FlowJo software (FlowJo LLC, USA).

### Degranulation assay

PBMC were incubated in CTL medium (IMDM medium, Life technologies^TM^ + 10 % fetal bovine serum, Invitrogen^TM^ + 10 Units/ml Penicillin / 10 μg/mL Streptomycin, Life technologies^TM^) at a ratio of 1:1 with K562 in the presence of anti-CD107a-PE/Cy5 for 3 h at 37 °C and 5 % (v/v) CO_2_. Cells were stained with CD3-BV510 and CD56-BV421 and then analyzed by flow cytometry. 

### ^51^Cr-release assay

K562 were labelled with 100 μCi ^51^Cr on a rotator for 1 h in a humidified incubator at 37 °C and 5 % (v/v) CO_2_. Co-incubation of PBMC and labelled K562 was performed in 96-well U-bottom plates and different effector to target ratios (6.5/1, 12.5/1, 25/1 and 50/1) for 4 h at 37 °C and 5 % (v/v) CO_2_. After incubation the supernatant was harvested and ^51^Cr release was analyzed with a γ-counter. Percent specific lysis was calculated as: 


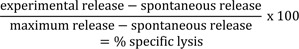


Since effector to target ratios are related to applied PBMC concentrations, the NK cell percentage of PBMC was used to recalculate the e/t ratio for NK cells in each individual sample. The number of lytic units (LU)/10^7^ was calculated for a lysis of 40 % as follows:


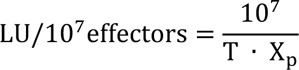


T: number of target cells, p: reference lysis level (here: 40 %), X_p_: e/t ratio required to lyse p% of target cells

### Cytometric bead array

To remove platelets and aggregates, coagulated blood was centrifuged and resulting serum was again centrifuged at 10,000 x g. Samples were stored at -80 °C until analysis. PBMC were stimulated for 6 h at 37 °C with 20 nM PMA (phorbol-12-myrisate-13-acetate, in DMSO, Cayman) and 2 μg/mL ionomycin (in DMSO, Calbiochem). The culture supernatant was stored at -80 °C before using the LEGENDplex^TM^ Human Inflammation Panel Kit according to the manufacturer's instructions. This kit allows the analysis of the 13 cytokines: IL-1β, IFN-α, IFN-γ, TNF-α, MCP-1 (CCL2), IL-6, IL-8 (CXCL8), IL-10, IL-12p70, IL-17A, IL-18, IL-23 and IL-33. Serum cytokine levels were measured using the same Kit. 

### Ferritin ELISA

Ferritin ELISA from IBL International GmbH was used according to the manufacturer's instructions to determine ferritin levels of the participant's serum samples. Plates were read by a microwell plate reader.

### Statistical analysis

Results from multiple experiments were reported as mean ± SD. Parameters for HH patients were compared to age-matched healthy controls. Significance levels were determined by the Wilcoxon rank-sum test (also known as Mann-Whitney U test, * *p* ≤ 0.05, ** *p* ≤ 0.01, *** *p* ≤ 0.001).

## Results

### Measurements of total cell numbers and immunophenotyping show minor variations for NK cells in treated HH patients

**Figure 1:**
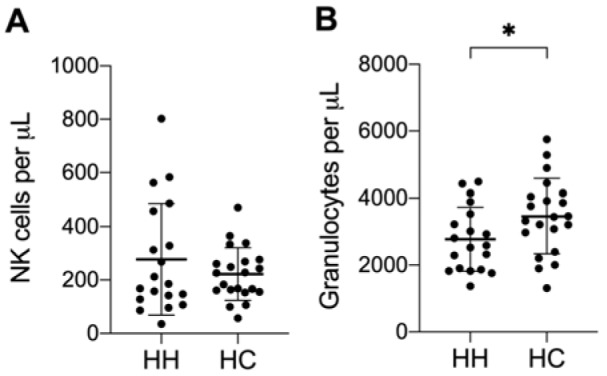
HH patients have fewer granulocytes in peripheral blood. Mean ± SD of absolute cell count (cells per μL blood) of A) NK cells and B) Granulocytes from 21 HH patients (HH) and healthy age-matched controls (HC). No significant changes were found for other leukocyte populations. The presented data is analyzed by Wilcoxon rank-sum test, also called Mann-Whitney U test: * indicates P<0.05 statistical significance.

Patients with hereditary hemochromatosis (HH) are affected by iron overload, which can lead to several consecutive symptoms. Connections between increased iron levels and immune cells were already shown (de Sousa et al., 1991[12]; Porto and De Sousa, 2007[24]). One of the treatment strategies for HH patients, to reduce systemic iron levels, is regular phlebotomy. Therefore, we wanted to investigate the immune cell composition, phenotype and function in treated HH patients. As some HH patients showed aberrant NK cell activities in our preliminary studies, we decided to focus on NK cells. We recruited 21 HH patients and 21 healthy controls for our study. First, we measured absolute cell numbers of leukocytes from whole blood samples via flow cytometry (TruCOUNT^TM^). We observed no changes in the absolute numbers of NK cells (Figure 1A(Fig. 1)), B cells, T cells, NK-T cells or monocytes (data not shown) in HH patients compared to healthy controls. However, HH patients demonstrated significantly lower granulocyte numbers compared to healthy controls (Figure 1B(Fig. 1)). 

**Figure 2:**
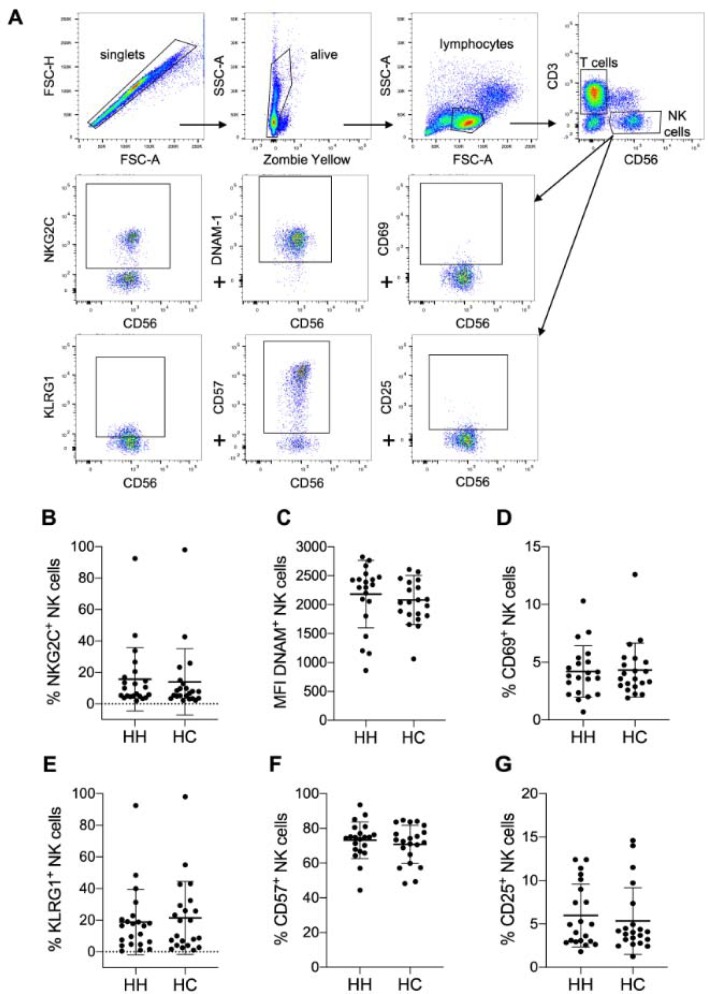
No altered expression of activation markers on NK cells of HH patients compared to controls. A) Gating strategy for measuring expression levels of selected NK cell activation markers by multicolor flow cytometry. Quantitative analysis of flow cytometry data for HH patients compared to healthy controls (each n=21): Mean ± SD of B) % positive NKG2C expressing NK cells, C) mean fluorescence intensity (MFI) of DNAM-1 positive NK cells, D) % CD69 NK cells, E) % KLRG1 positive NK cells, F) % CD57 positive NK cells and G) CD25 positive NK cells. The data is analyzed by Wilcoxon rank-sum test, also called Mann-Whitney U test; no significant variation between HH and HC was observed.

**Figure 3:**
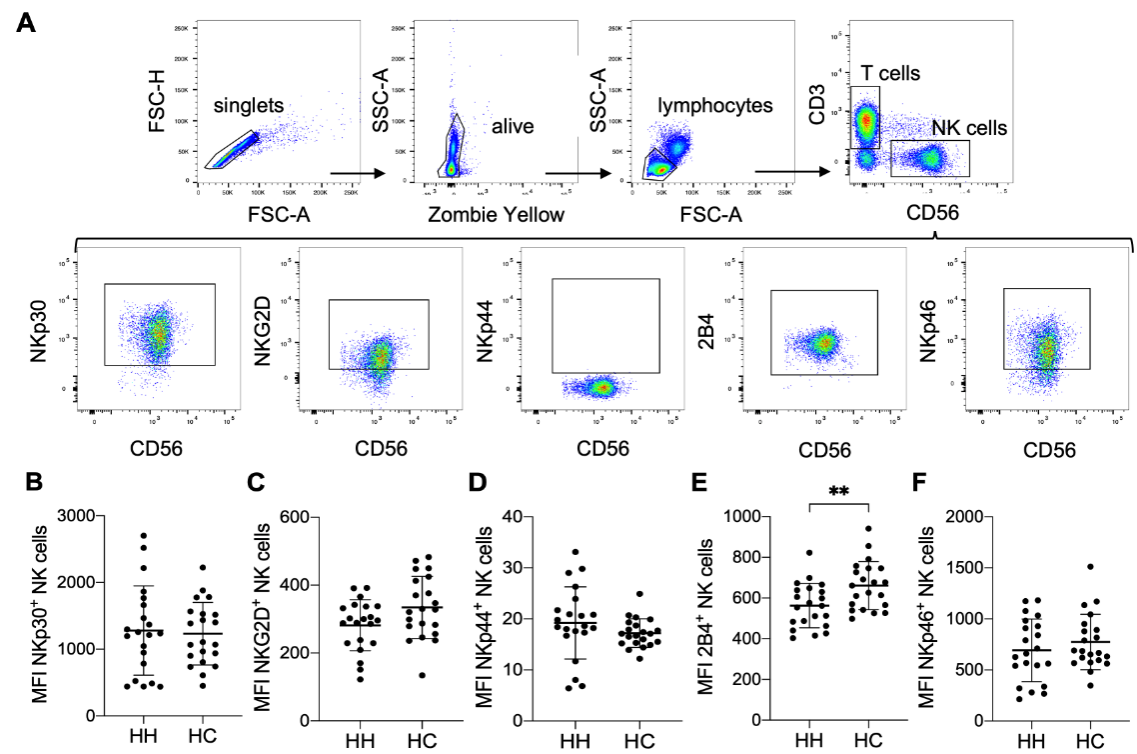
Lower expression levels of the activating receptor 2B4 on NK cells of HH patients. A) Gating strategy for measuring expression levels of selected NK cell activation receptors by multicolor flow cytometry. Quantitative analysis of flow cytometry data for HH patients compared to healthy controls (each n=21): Mean ± SD of B) mean fluorescence intensity (MFI) of NKp30 positive NK cells, C) MFI of NKG2D positive NK cells, D) MFI of NKp44 positive NK cells, E) MFI of 2B4 positive NK cells and F) MFI of NKp46 positive NK cells. The data is analyzed by Wilcoxon rank-sum test, also called Mann-Whitney U test; ** indicates P<0.01 statistical significance.

To analyze the phenotype to NK cells, we designed three multicolor cytometry panels (Table 1(Tab. 1)): (i) A leukocyte panel for a general overview of PBMC subsets and their distribution, (ii) an activation panel for expression levels of selected NK cell activation and memory markers and (iii) a receptor panel for the NK cell activation receptors NKp30, NKp44, NKp46, 2B4 and NKG2D. Frozen PBMCs of HH patients and healthy controls were thawed, stained and directly analyzed by flow cytometry. For leukocyte phenotyping, dead cells were first excluded by zombie yellow staining and leukocytes were identified by gating on CD45 positive cells. Quantification of NK cell, T cell and B cell frequencies of PBMC as well as of CD14 positive monocytes showed comparable results for treated HH patients and healthy controls (Supplementary Figure 1). Similarly, NK cells showed no differences in the expression of activation and differentiation markers such as NKG2C, DNAM-1, CD69, KLRG1, CD57 or CD25 when comparing treated HH patients and healthy controls (Figure 2(Fig. 2)). Finally, we did not find statistically significant differences in the expression levels of the activating NK cell receptors NKp30, NKp44, NKp46, or NKG2D between treated HH patients and healthy controls (Figure 3(Fig. 3)). In contrast, the activating receptor 2B4 showed a significantly lower expression (p<0.01) on NK cells of treated HH patients compared to healthy controls (Figure 3E(Fig. 3)).

### NK cells of hemochromatosis patients show slightly higher cytotoxicity

**Figure 4:**
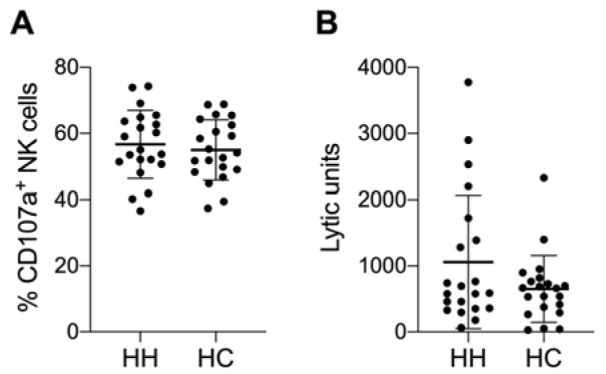
Slightly increased NK cell cytotoxicity in some HH patients. Summarized data (mean ± SD) of A) CD107a positive NK cells after 3 h incubation with K562 as target cells and B) of lytic units per 10^7^ effectors with a specific lysis of 40 % of chromium release assays with K562 as target cells after 4 h incubation. The data is analyzed by Wilcoxon rank-sum test, also called Mann-Whitney U test; no significant variations between hereditary hemochromatosis patients (HH) and healthy controls (HC) was observed. N=21 for HH and HC respectively.

Next we tested the functional activity of NK cells. NK cells release their cytotoxic granules upon the killing of a sensitive target cell. This degranulation can be measured by detecting the externalization of CD107a on the surface of NK cells. PBMCs were co-cultured with K562 target cells for 3 h and the number of degranulating NK cells (CD107a positive NK cells) was determined by flow cytometry. While we clearly observed the degranulation of NK cells, there were no differences between treated HH patients and healthy controls (Figure 4A(Fig. 4)). Additionally, the NK cell-mediated killing of target cells can be measured using a chromium release assay. Therefore, PBMCs were incubated with ^51^Cr-labeled K562 target cells for 4 h and the lytic activity of NK cells was calculated. Although some treated HH patients showed higher NK cell cytotoxicity compared to controls (Figure 4B(Fig. 4)), there were no statistically significant differences in the cytotoxic activity of NK cells comparing HH patients to healthy controls.

### Increased pro-inflammatory cytokine production by PBMC of treated HH patients

**Figure 5:**
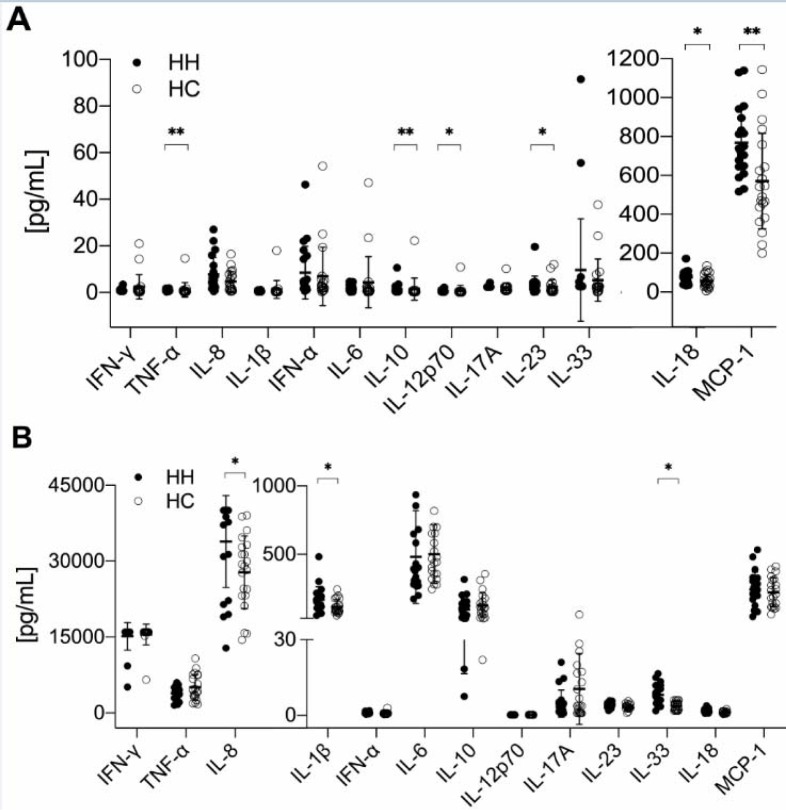
Increased pro-inflammatory cytokine production by PBMCs of HH patients. Mean ± SD of 13 different cytokines; A) concentrations of examined serum samples and B) produced by PBMC after unspecific stimulation with ionomycin and PMA (phorbol-12-myristate-13-acetate). The data is analyzed by Wilcoxon rank-sum test, also called Mann-Whitney U test; n=21 for HH and HC respectively. * indicates P<0.05 statistical significance.

Cytokines are important for the regulation of immune reactions and they are involved in many immune-mediated diseases. Therefore, we measured the concentration of 13 different cytokines in the serum of HH patients and healthy controls. Cytokine concentrations of HH patients were slightly, but significantly lower in case of TNF-α (p<0.01), IL-10 (p<0.01) and IL-12p70 (p<0.05), and slightly higher for MCP-1 (p<0.01), IL-18 (p<0.05) and IL-23 (p<0.05) (Figure 5A(Fig. 5)). Additionally, we measured the cytokine concentration produced by PBMC after their stimulation with PMA and ionomycin. Here we observed an increased production of pro-inflammatory cytokines in treated HH patients: IL-1β (p<0.05), IL-8 (0.01), IL-18 (p<0.01) and IL-33 (p<0.001) compared to healthy controls (Figure 5B(Fig. 5)).

### Well-adjusted serum ferritin levels of treated hemochromatosis patients

**Figure 6:**
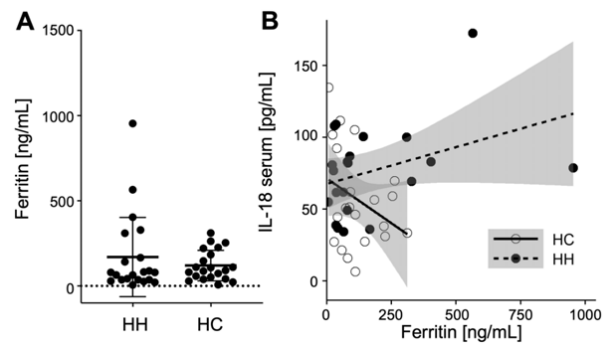
Normal ferritin levels and no correlation with pro-inflammatory cytokine concentrations in HH patients. A) Summarized data of ferritin serum concentrations. The data is analyzed by Wilcoxon rank-sum test, also called Mann-Whitney U test; no significant variations between hereditary hemochromatosis patients (HH) and healthy controls (HC) was observed. N=21 for HH and HC respectively. B) Correlation of IL-18 serum concentrations with ferritin serum concentrations for HH and HC (data for remaining cytokines not shown).

Finally, we measured serum ferritin levels to determine potential relationships with the immune parameters. We found that, except for a few outliers, all HH patients showed ferritin levels comparable to the healthy control group (Figure 6A(Fig. 6)). Therefore, the treatment of the HH patients seemed to be effective in keeping their iron levels within normal parameters. Next we wanted to test if there is a correlation between the variations in immune parameters of HH patients and their ferritin levels. While we observed a highly variable expression of IL-18 in serum in HH patients as well as in healthy controls, these were not correlated to ferritin levels of the subjects (Figure 6B(Fig. 6)). Also, neither the higher levels of ferritin in some treated HH patients, nor the higher expression levels of some cytokines correlated with the increased cytotoxicity of outliers in the chromium release assay (data not shown).

## Discussion

Based on previous observations in our lab, we wanted to examine whether selected immune cells, especially NK cells, are phenotypically and functionally influenced in treated hemochromatosis patients. Therefore, blood samples of twenty-one hereditary hemochromatosis patients and age-matched healthy controls were studied. 

With the exception of granulocytes, we did not find significant differences in absolute immune cell numbers or the relative distribution of PBMC subpopulations for HH patients compared to the healthy control group. It was reported that iron overload or high serum ferritin levels are associated with low lymphocyte counts regardless of whether hereditary hemochromatosis patients exhibit the HFE mutation C282Y (homo- or heterozygote) or C282Y/H63D compound heterozygote (Barton et al., 2005[6]). However, most of our tested HH patients were medically well adjusted by phlebotomy to treat iron overload as evident by the normal serum ferritin concentrations in most HH patients. This may explain why we did not find major differences in lymphocyte numbers. Several studies found a correlation between iron overload and a reduced phagocytic and bactericidal activity of neutrophils (Boelaert et al., 1990[7]; Cantinieaux et al., 1988[10]; Patruta et al., 1998[22]; van Asbeck et al., 1984[30]). While this may be related to our finding that HH patients have lower absolute numbers of granulocytes, further studies are warranted to investigate this finding in more detail. 

Regular phlebotomy not only prevents iron overload in HH patients, but it also results in repeated immune cell loss. Interestingly, this does not seem to affect immune cell numbers in HH patients, indicating that the loss of immune cells due to phlebotomy is rapidly counteracted by de-novo generation. 

Our phenotypic analysis did not find any major differences in NK cells between HH patients and healthy controls. We only found a lower expression of the activating receptor 2B4. However, this reduction of 2B4 expression does not seem to have functional consequences for NK cell cytotoxicity, as we found normal NK cell degranulation and cytotoxicity in HH patients. The reason for the lower expression of 2B4 is unknown. The genotype of patients could play a role, but we found no data in the literature describing such an effect.

Cytokine levels in serum or after stimulation of HH patient's PBMC showed a tendency for increased production of pro-inflammatory cytokines. Iron and its homeostasis are already known to be influenced through distinct inflammatory cytokines by regulating ferritin translation (Torti and Torti, 2002[29]; Wessling-Resnick, 2010[34]). Further, a correlation between inflammatory cytokines and hepcidin is described in iron deficiency anemia (IDA). Here, increased levels of pro-inflammatory cytokines lead to increased hepcidin synthesis which blocks the iron efflux from cells resulting in lower iron levels. Especially higher levels of the pro-inflammatory cytokines TNF-α and IL-6 are described to lead to increased hepcidin levels, causing IDA (Askar et al., 2019[2]; Atkinson et al., 2008[4]; Ganz, 2003[17]; Ganz and Nemeth, 2012[18]). Conversely, monocytes of HFE associated hemochromatosis patients produce less TNF-α *in vitro* (Gordeuk et al., 1992[19]). This converse effect was also shown in a study for IL-6, in which healthy adults displayed a strong reduction of serum iron upon receiving an IL-6 infusion (Nemeth et al., 2004[21]). IL-6 was also described to be able to increase hepcidin synthesis (Askar et al., 2017[3]). These findings rise the question of causality between these pro-inflammatory cytokines and systemic iron levels. In our study, we found significantly lower production of TNF-α and IL-6 in treated HH patients, but we are not able to distinguish whether cytokines influence iron levels or vice versa. Loss of HFE function for example already results in reduced hepcidin levels and increased ferroportin levels, while lowering iron levels in monocytes and macrophages (Cairo et al., 1997[9]). Again, the HFE gene itself seems to have an influence on regulatory mechanisms of immune cells, leading to different production repertoires of cytokines, which promote altered iron levels. This would also explain why there is no obvious correlation between the cytokine production and ferritin levels, since the patients are treated to get rid of the iron overload but still produce altered repertoires of cytokines caused by their genotype. Comparable to our results, IL-1β production by monocytes was not significantly different between HH patients and the control group described by Gordeuk et al. (1992[19]). Increased levels of the remaining pro-inflammatory cytokines or IL-10 have not yet been associated with iron overload or hemochromatosis but could explain the slightly increased NK cell cytotoxicity. 

In summary, we did not find major phenotypic or functional alterations in NK cells and other immune cells in HH patients, suggesting that if the disease is treated there are no major impacts on the immune system. 

## Acknowledgements

We thank all the volunteers who participated in this study. 

## Conflict of interest

The authors declare that they have no conflict of interest.

## Supplementary Material

Supplementary information

## Figures and Tables

**Table 1:**
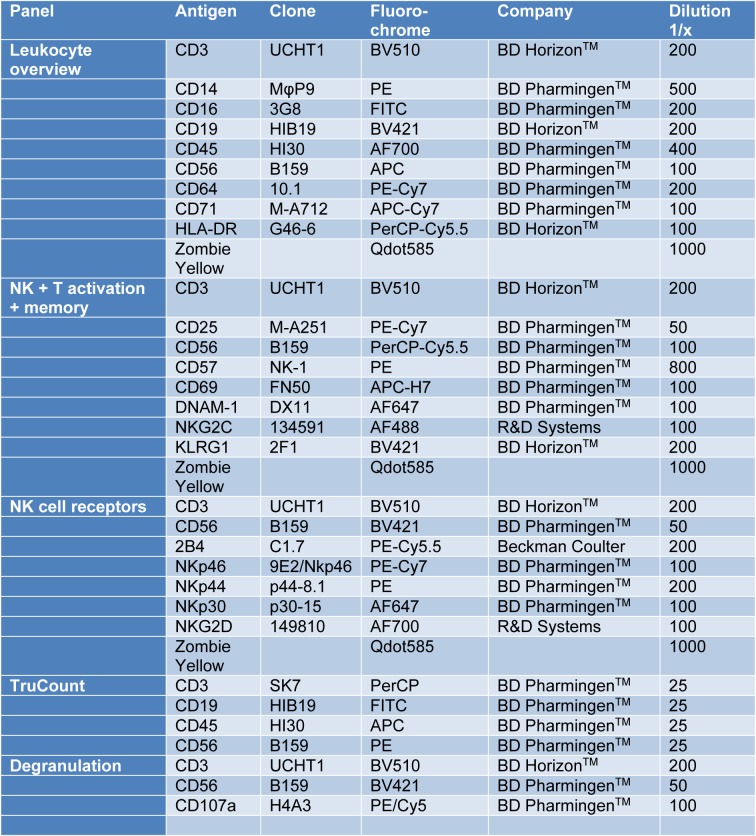
Panel description, antigens, antibody clones and coupled fluorochromes, distributors and Ab dilution used to stain 0.2 × 10^6^ PBMC are listed.
