# Supplementary material for: Analysis of Natural Killer cell functions in patients with hereditary hemochromatosis
Source: EXCLI J. 2020 Mar 25;19:430–41. doi: 10.17179/excli2020-1116 (PMC7174573; doi:10.17179/excli2020-1116)
Supplement: Supplementary information [file EXCLI-19-430-s-001.pdf]

EXCLI Journal 2020;19:430-441 – ISSN 1611-2156
Received: February 04, 2020, accepted: March 23, 2020, published: March 25, 2020

Supplementary information to:

ANALYSIS OF NATURAL KILLER CELL FUNCTIONS IN PATIENTS WITH HEREDITARY HEMOCHROMATOSIS

Vivian Bönnemann[1], Maren Claus[1], Barbara Butzeck[2], Daniela Collette[3], Peter Bröde[1], Klaus Golka[1], Carsten Watzl[1]

[1] Department for Immunology, Leibniz Research Centre for Working Environment and Human Factors (IfADo) at TU Dortmund, Dortmund, Germany
[2] Hämochromatose-Vereinigung Deutschland e.V. HVD, European Federation of Associations of Patients with Haemochromatosis EFAPH, Hattingen, Germany
[3] Gemeinschaftspraxis für Hämatologie und Onkologie, Dortmund, Germany

* **Corresponding author:** Carsten Watzl, Leibniz Research Centre for Working Environment and Human Factors (IfADo) at TU Dortmund, Ardeystrasse 67, 44139 Dortmund, Germany, E-mail: watzl@ifado.de

http://dx.doi.org/10.17179/excli2020-1116

This is an Open Access article distributed under the terms of the Creative Commons Attribution License (http://creativecommons.org/licenses/by/4.0/).

EXCLI Journal 2020;19:430-441 – ISSN 1611-2156
Received: February 04, 2020, accepted: March 23, 2020, published: March 25, 2020

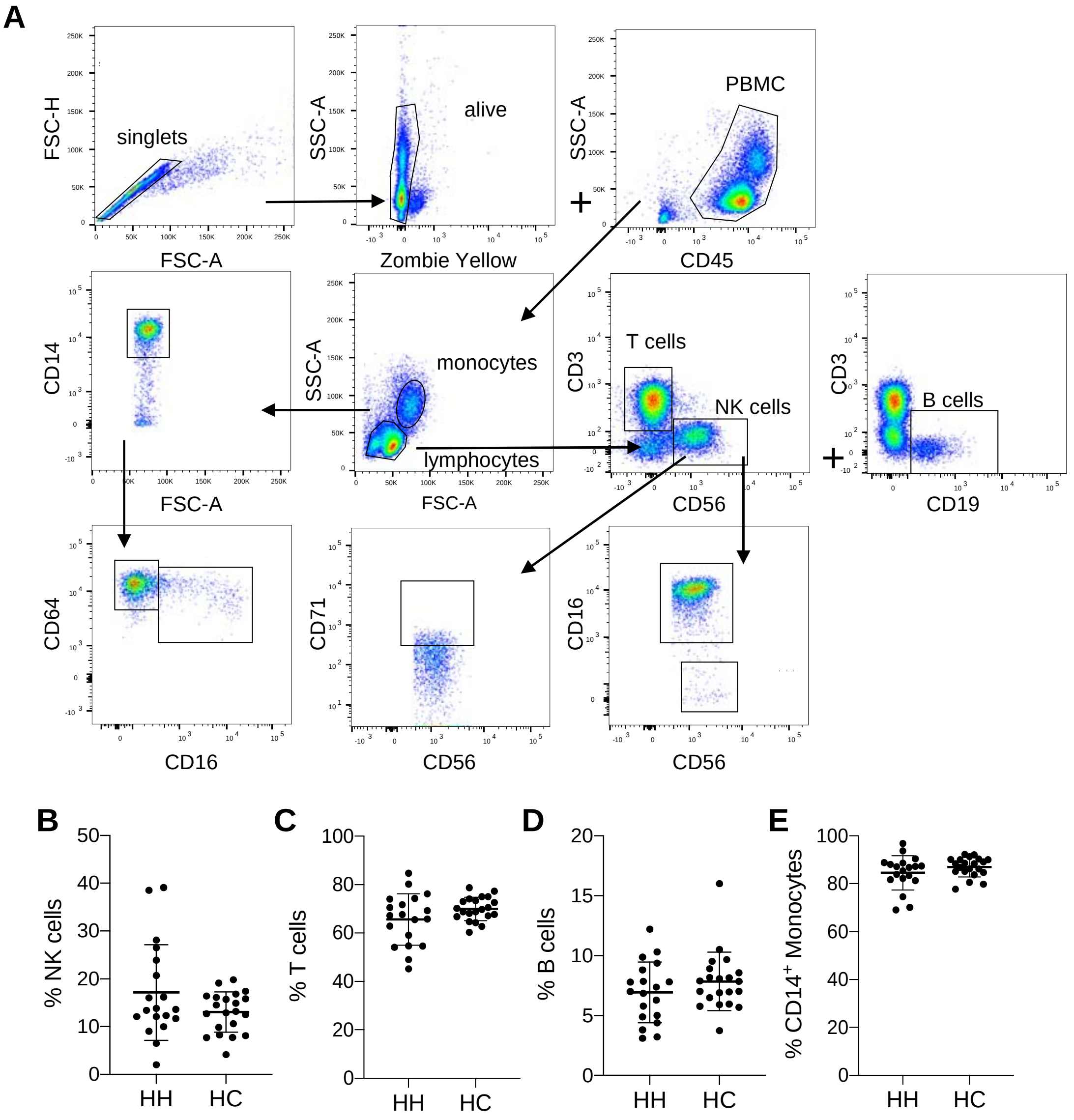

Supplementary Figure 1: Analysis of leukocyte subpopulations by multicolor flow cytometry. A) Gating strategy for a general overview of lymphocyte and monocyte subpopulations. **B**) Quantitative analysis of flow cytometry data for HH patients compared to healthy controls (each n=21): Mean ± SD of **B** % NK cells of lymphocytes, **C** % T cells of lymphocytes, **D** % B cells of lymphocytes, **E** % CD14 positive monocytes of monocytes (in PBMC). The data is analyzed by Wilcoxon rank-sum test, also called Mann-Whitney U test; no statistical significance was detected.
